# Dynamic Stability Enhancement of Columns Through Material Distribution Optimization Strategies

**DOI:** 10.3390/ma18102167

**Published:** 2025-05-08

**Authors:** Janusz Szmidla, Anna Jurczyńska, Robert Ulewicz

**Affiliations:** 1 Faculty of Mechanical Engineering, Czestochowa University of Technology, 42-201 Czestochowa, Poland; janusz.szmidla@pcz.pl (J.S.); robert.ulewicz@pcz.pl (R.U.); 2 Faculty of Management, Czestochowa University of Technology, 42-201 Czestochowa, Poland

**Keywords:** bridge span, optimal shaping, non-prismatic column, buckling, critical force

## Abstract

This study focuses on optimizing the shape of columns, particularly considering advanced material distributions that respond to specific load cases. Utilizing a variational method, equations describing movement and boundary conditions are established. This research, while adhering to a static and kinetic criterion for stability loss, identifies the optimal geometric parameters for the columns constructed from specific materials to achieve maximum critical load capacity. It is assumed that the total volume of the system does not change. The innovation of the presented research is the use of a simulated annealing algorithm to optimally shape the column outline in terms of the maximum critical force value, which depends on many material variables. This method was adapted to the calculations of slender rod systems by introducing a number of modifications. The obtained increases in the critical load reach up to 40% compared to the prismatic system. The results also show that it is possible to control the dynamic properties in column structures while increasing the stability of the system. This study underscores the significant role of material selection and optimization in enhancing the dynamic stability and load-bearing capacity of column structures.

## 1. Introduction

One of the most popular scientific problems these days is the issue of stability and free vibrations in slender systems. Optimizing systems by reducing the mass while simultaneously increasing the strength properties of the systems, including improving dynamic properties, is one of the biggest challenges facing scientists. Among the methods used to achieve this goal, the following should be mentioned: the pre-compression of systems, taking into account additional elements in models (elastic support or piezoceramic elements, linear or rotational springs, or dampers), new ideas for implementing the load, and shape optimization. In addition, there is a significant increase in the popularity of using advanced, multilayer composite or composite improved with additive materials, whose variable mechanical properties are often described by complex nonlinear equations.

Currently, scientific’ research focuses on improving the strength properties of structures. Another aspect of the work being carried out is the ability to control their dynamic properties. One of the guarantees that a machine or structure will work properly and will not fail prematurely is correctly performed static and dynamic studies, including accurate reproductions of the model and materials used. Among the aspects discussed in works on the optimization and improvement of the dynamic properties of slender systems, the most frequently mentioned are as follows: the most accurate mathematical description of the model and material, additional elements in physical models, and optimal shaping of the system.

In terms of realizing mathematical models, physical (see refs. [1,2,3,4]) and geometric (see refs. [1,5,6,7,8]) nonlinearities, inhomogeneities, friction [1], and damping are taken into account in considerations. An analysis of nonlinear beams supported on linear elastic foundations under harmonic excitation was discussed in paper [6]. Through the presented numerical results, it has been shown that the stretching potential energy is responsible for generating the cubic nonlinearity in the considered system. In article [7], a buckling resistance examination of a rod structure made of steel was studied. The authors used the finite element method to obtain results from a comprehensive numerical study. The geometrically nonlinear column subjected to the selected case of the specific Tomski’s load was investigated in article [8], in terms of limiting the range of local instability. It has been proved that proper shape optimization of the non-prismatic rod as a component of the system may cause an increase in bifurcation load. Research on nonlinear vibrations and a postbuckling analysis of beams are presented in [2]. The authors assumed that analyzed systems under an axial force are made of functionally graded materials, lying on a nonlinear elastic base. The Galerkin method and the variational iteration method were used to obtain numerical results. Similar scientific problems were also discussed in papers [3,4]. In article [9], a new solution to the stability issue of columns of variable cross-sections under axial forces was described. The proposed approach consists in the double division of a column into segments. Each of the parts from the first fragmentation are replaced with a rigid element understood as a joint with springs: translational and rotating. The authors analyzed double symmetrical rods with dimensions changing continuously and abruptly. In paper [10], an explicit formula for the critical load value of columns subjected to the non-uniform distribution of axial forces was presented. Based on the Euler’s formula on the buckling load, a significant number of charts defining the value of the correction coefficient, taking into consideration the size of the cross-section, the type of convergence, and loading, was shown. The maximum recorded difference between the described approach and numerical calculations did not exceed 7%. The paper [11] concerns the analysis of a concrete beam, made of material that is both geometrically and materially nonlinear. In the formulation of the problems, the Hellinger–Reissner variational principle is used. Similar problems can be found in paper [12], where a cylindrical main plate was subjected to tests. The authors checked the influence of plate thickness and material nonlinearities on the stability of the system. The topic of nonlinear mechanical–thermal analysis using reinforced, functionally graded porous material is included in paper [13]; similar findings to the material research are presented in papers [14,15,16]. In paper [17], a solution to the problem of vibrations in beams made of graphene-reinforced porous nanocomposite, which was subjected to moving loads, is shown. As a method of reducing vibrations, an inertial nonlinear energy absorber (NES) was used for the first time.

Another way to implement the studied models is to include additional elements in the systems, e.g., elastic or piezoceramic elements (see [18]), to support the systems with elastic foundations or springs, or using oscillators (e.g., in [19]). All these elements are designed to reflect the way the system is mounted and loaded. In paper [20], the issue of stability in slender systems subjected to Euler’s force, with consideration of Timoshenko’s theory, is presented. Different values of slenderness factor, as well as translational and rotational spring stiffness, were discussed through the obtained results. Similar results were described in Ref. [21], where the boundary problem of stability and free vibrations in a slender elastic system in the form of a hydraulic cylinder under Euler’s load was examined. In article [22], the authors discuss the usefulness of a piezoceramic rod discretely mounted to a host column for the purpose of suppressing the lateral deflection, assuming eccentric load application. It has been proved that the piezoactuation realized by considering the piezoceramic rod is a very efficient and powerful way of controlling the shape of the analyzed type of structure. The problem of the stability of a geometrically nonlinear column was presented in paper [23], where pre-stressing and partial support on a Winkler elastic foundation was also taken into account. The authors analyzed the phenomenon of local loss of rectilinear form in static equilibrium. A closed-form solution to the moving harmonic loading issue for continuous beam systems (defined using Euler–Bernoulli theory) was addressed in article [24]. It has been proven that it is possible to find the solution to the harmonic component of any load spectrum. In paper [25], the solution for the eigenforms of a beam on elastic support was presented. Ref. [5] considers a coupled system of smooth and discontinuous absorbers and a beam bridge subjected to moving loads. It was designed to measure the efficiency of the smooth and discontinuous absorbers. In paper [26], a nonlinear analysis of the dynamic response of a complex structure was presented using the mode weighting method.

The issue of stability was also analyzed by Q. S. Li [27,28]. In his research, he examined systems with abrupt and constant changes in dimension along the axis. The influence of different external load case (conservative or follower), mounting type (also support by springs), or system geometry on the value of the buckling load was included in his studies. In the papers mentioned, the finite element method and the transfer matrix method were used to obtain the solution of the boundary issues under consideration.

Another group of publications are those containing analyses of the possibilities of optimizing the system using available algorithms [9,29]. A rigid multibody approach used for the analysis of non-prismatic column buckling was proposed in paper [9]. The influence of the selfweight characteristics of springs at the ends of the column, and axial compressive intermediate force, on the critical forces of the system was discussed through presented numerical results. In article [29], the results of complex studies relating to the shape optimization of columns subjected to follower force, directed towards the pole, were presented. In the paper [30], the optimization of the truss shape was included using millions of failure sequences. The problems of buckling, free vibrations, and modal analysis in non-prismatic columns were presented in paper [31]. Optimized shape functions and the Raileigh method were used to formulate the problem. The optimization problem is also the subject of paper [32], but it concerns the plate, and the considerations additionally include applications in cellular sandwich structures.

The multitude of scientific works in this area shows how important the above-mentioned factors are for the structure. Changing the outline of the structural components significantly affects the strength properties. It was also proven that it can limit costs by reducing the weight of the system. Even more accurate studies of the system’s behavior during actual operation are possible, and can be performed by including nonlinearity in mathematical and physical models, which increases the quality of the representation of the actual system behavior. Engineering practice shows that the significance of stability and vibration problems in slender elastic systems is gaining increasing importance in recent years, and has attracted more attention in many disciplines, especially in mechanical engineering, aviation, and construction. Structures like those under consideration in this article are commonly used as real solutions: in bridge constructions [33,34,35,36,37] or supporting structures in civil engineering.

Optimization issues include many methods that can be easily adapted to the engineering problems being undertaken. One such method is the simulated annealing method, which is used in various fields of technology [38,39,40]. Paper [41] describes the application of the simulated annealing method to the multi-criteria optimization of the design of a flat aluminum radiator. Two objective functions are considered independently and together; the first is the minimization of the temperature on the base plate, and the second is the reduction in the radiator volume. Based on the presented results, new possibilities for creating technical solutions for radiators with reduced weight and improved thermal conductivity have been demonstrated. Paper [42] shows how the simulated annealing algorithm can be used to effectively optimize the geometry of three-dimensional structures. Similar research results can be found in paper [43], where simulated annealing is presented as a new method for increasing the developability of a quasi-developable Bézier surface from two design curves. This method has also been used in the development of a reliable charging schedule for electric vehicle batteries [44].

This paper deals with the problem of free vibrations and stability in a geometrically nonlinear column where some element has variable material distribution, modeling the bridge span. The studies discussed focus on the change in the bifurcation load and free vibrations. The outline of the rod with a variable material distribution is subjected to optimal shaping, taking advantage of a modified simulated annealing algorithm. The introduced modifications concern, among other factors, the operation of the function generating the neighboring solution (neighborhood function), the selection of a new shape of the optimized system while maintaining the criterion of constant volume, and the number of segments n of the column variable in geometric progression, simplifying the search for the optimal outline. In order to verify the correctness of the adopted mathematical models, and the obtained results from numerical calculations, experimental studies are carried out on the changes in the first three natural frequencies.

## 2. Physical Model

**Figure 1:**
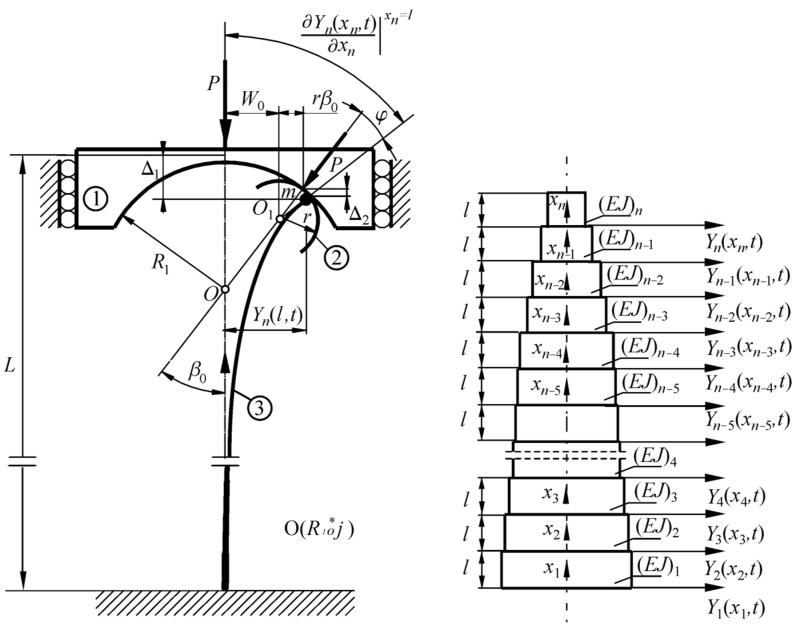
The scheme of the considered column subjected to the follower force directed towards the pole (on the **left**). The method of modeling a rod with a variable cross-section (on the **right**).

The physical model of the column under consideration subjected to load acting through the loading ➀ and receiving heads ➁ with a circular outline (the curvature is assumed to be constant) is presented in Figure 1. The external load direction goes through the point *O* located on the undeformed axis of the structure. This type of conservative load is called follower force, directed towards the positive pole. The receiving load heads are assumed to be infinitely rigid. The rods ➂ are rigidly mounted on one end (*x*_1_ = 0), and they are connected at the second end (*x_n_* = *l*) to the loading system via an element, the concentrated mass of which was taken into account as a mass *m*.

To simplify the description of the problem, the column under consideration (Figure 1) is divided into smaller prismatic parts (subscripts *i* = 1, 2, …, *n*) of a circular cross-section: compressive stiffness (ρ*A_i_*) and flexural stiffness (*EJ_i_*), length *l*, and diameter *d*, described by transverse displacement Y*_i_*(*x_i_,t*). The optimal shaping is based on the selection of the dimensions of the particular parts at which the maximum buckling load value can be obtained. Thanks to this assumption, the authors searched for the maximum buckling force value, which may have depended on multiple variables, i.e., the dimensions of particular column parts. In a generalized form, the function describing the value of the critical force can be written as follows:

(1) $$\;\;P_{max}=f(d_{1},\;d_{2},\ldots ,d_{n},\frac{L}{n}).$$

The buckling force value *P_kr_* of the analyzed rods of variable cross-section was related to the flexural stiffness (*EJ*)*_pr_* of comparative structures, which is assumed to be constant along the length *L*:

(2) $$\lambda_{oc}=\frac{P_{kr}L^{2}}{{\left(EJ\right)}_{pr}}.$$

In the problem formulation, it was assumed that the total length *L*, the volume of the whole column *V* and the value of Young’s modulus *E* are constant for both optimized and comparative (prismatic) columns:

(3) n⋅l=L=idem,

(4) $$\sum_{i=1}^{n}v_{i}=V=idem,$$

where *v_i_*—the volume of the *i*-th segment, and *V*—the total volume of the column.

This chapter implements the following designations of the systems under consideration:

- O(*R*_1_*)—the system under the follower force directed towards the positive pole with a variable material distribution, with the given parameter of the loading and receiving head radius *R*_1_* that is subjected to the optimal shaping;
- P(*R*_1_*)—a comparative column with flexural stiffness (*EJ*)*_pr_* constant along the length of the system, loaded by a follower force directed towards the positive pole, with the loading head parameter *R*_1_*.

## 3. Boundary Problem Formulation

The equations of motion and boundary conditions of the columns under consideration were determined based on Hamilton’s principle

(5) $$\delta \int_{t_{1}}^{t_{2}}\left(T-\sum_{\xi =1}^{3}V_{\xi }\right)dt=0,$$

where *T* is the kinetic energy, *V_ζ_* is the potential energy, *t* is time, and *δ* is the operator of variations.

The kinetic energy *T* of the columns under consideration consist of the kinetic energy of the particular segments and the kinetic energy of the mass *m*

(6) $$T=\sum_{i=1}^{n}\frac{\left(\rho A_{i}\right)}{2}\int_{0}^{l}{\left[\frac{\partial Y_{i}\left(x_{i},t\right)}{\partial t}\right]}^{2}dx_{i}+\frac{m}{2}{\left[\frac{\partial Y_{n}\left(l,t\right)}{\partial t}\right]}^{2},$$

where *A_i_* is the area of cross-section of *i*-th segment of the analyzed column.

The potential energy constituents were defined as follows:

- the energy of bending elasticity:

(7) $$V_{1}=\sum_{i=1}^{n}\frac{\left(EJ_{i}\right)}{2}\int_{0}^{l}{\left[\frac{\partial^{2}Y_{i}\left(x_{i},t\right)}{\partial {x_{i}}^{2}}\right]}^{2}dx_{i},$$

- the energy of the vertical component of the force *P*:

(8) $$V_{2}=-\sum_{i=1}^{n}\frac{P}{2}\int_{0}^{l}{\left[\frac{\partial Y_{i}\left(x_{i},t\right)}{\partial x_{i}}\right]}^{2}dx_{i},$$

- the energy of the horizontal component of the force *P*:

(9) $$V_{3}=\frac{P}{2}R_{1}{\left[{\left.\frac{\partial Y_{n}\left(x_{n},t\right)}{\partial x_{n}}\right|}^{x_{n}=l}\right]}^{2}.$$

The geometric boundary conditions known a priori for the problem under consideration are as follows:

- At the attachment point (*x*_1_ = 0)

(10) $$Y_{1}\left(0,t\right)={\left.\frac{\partial Y_{1}\left(x_{1},t\right)}{\partial x_{1}}\right|}_{x_{1}=0}=0;$$

- The continuity conditions

(11) $$Y_{\zeta }\left(l,t\right)=Y_{\zeta +1}\left(0,t\right),{\left.\frac{\partial Y_{\zeta }\left(x_{\zeta },t\right)}{\partial x_{\zeta }}\right|}_{x_{\zeta }=l}={\left.\frac{\partial Y_{\zeta +1}\left(x_{\zeta +1},t\right)}{\partial x_{\zeta +1}}\right|}_{x_{\zeta +1}=0},$$

where *ζ*= 1, …, (*n* − 1).

By applying the geometrical relationships between the elements of the loading structure, the following was obtained:

(12) $$Y_{n}\left(l,t\right)=R_{1}{\left.\frac{\partial Y_{n}\left(x_{n},t\right)}{\partial x_{n}}\right|}^{x_{n}=l}.$$

Taking into account the formula describing Hamilton’s principle (5), after the application of appropriate boundary conditions (10)–(12), and performing some algebraic transformations, the following were determined:

- Differential equations of motion for the particular segments of the rod

(13) $$\left(EJ_{i}\right)\frac{\partial^{4}Y_{i}\left(x_{i},t\right)}{\partial {x_{i}}^{4}}+P\frac{\partial^{2}Y_{i}\left(x_{i},t\right)}{\partial {x_{i}}^{2}}+\left(\rho A_{i}\right)\frac{\partial^{2}Y_{i}\left(x_{i},t\right)}{\partial t^{2}}=0,\;\;\;i=1,\;2,\;\ldots ,\;n$$

- Missing natural continuity conditions and natural boundary conditions at the free end (*x_n_* = *l*)

(14) $${\left.\frac{\partial^{2}Y_{\zeta }\left(x_{\zeta },t\right)}{\partial {x_{\zeta }}^{2}}\right|}_{x_{\zeta }=l}={\left.\;\kappa_{\zeta +1}\;\frac{\partial^{2}Y_{\zeta +1}\left(x_{\zeta +1},t\right)}{\partial {x_{\zeta +1}}^{2}}\right|}_{x_{\zeta +1}=0},$$

(15) $${\left.\frac{\partial^{3}Y_{\zeta }\left(x_{\zeta },t\right)}{\partial {x_{\zeta }}^{3}}\right|}_{x_{\zeta }=l}={\left.\;\kappa_{\zeta +1}\;\frac{\partial^{3}Y_{\zeta +1}\left(x_{\zeta +1},t\right)}{\partial {x_{\zeta +1}}^{3}}\right|}_{x_{\zeta +1}=0},$$

(16) $${\left.\frac{\partial^{3}Y_{n}\left(x_{n},t\right)}{\partial {x_{n}}^{3}}\right|}_{x_{n}=l}-\frac{1}{R_{1}}{\left.\frac{\partial^{2}Y_{n}\left(x_{n},t\right)}{\partial {x_{n}}^{2}}\right|}_{x_{n}=l}-\frac{m}{\left(EJ_{n}\right)}\frac{\partial^{2}Y_{n}\left(l,t\right)}{\partial t^{2}}=0,$$

where ${k_{n}}^{2}=P/\left(EJ_{n}\right),\kappa_{\zeta +1}=\left(EJ_{\zeta +1}\right)/\left(EJ_{\zeta }\right).$

## 4. Solution of Boundary Issue (Energetic Method)

Given the constant volume criterion, conducting optimization studies requires knowledge of the value of the buckling load. The static stability criterion (energetic approach) was used to determine the buckling load parameter. The problem is formulated based on the condition of minimum total potential energy given in the following form:

(17) $$\delta \sum_{\xi =1}^{3}V_{\xi }=0.$$

After the previous separation of variables of the function Y*_i_*(*x_i_,t*), in relation to time *t* and spatial coordinates *x_i_*, the potential energy of the column (Equations (7)–(9)) is as follows:

(18) $$Y_{i}\left(x_{i},t\right)=y_{i}\left(x_{i}\right)\cos\left(\omega t\right)$$

The boundary conditions (10)–(12), (14)–(16) after applying Formula (18) are as follows:

(19) $$y_{1}\left(0\right)=y_{1}^{I}\left(0\right)=0,$$

(20) $$y_{n}\left(l\right)=R_{1}y_{n}^{I}\left(l\right),$$

(21) $$y_{n}^{III}\left(l\right)-\frac{1}{R_{1}}y_{n}^{II}\left(l\right)=0,$$

(22) $$y_{\zeta }\left(l\right)=y_{\zeta +1}\left(0\right),$$

(23) $$y_{\zeta }^{I}\left(l\right)=y_{\zeta +1}^{I}\left(0\right),$$

(24) $$y_{\zeta }^{II}\left(l\right)=\kappa_{\zeta +1}y_{\zeta +1}^{II}\left(0\right),$$

(25) $$y_{\zeta }^{III}\left(l\right)=\kappa_{\zeta +1}y_{\zeta +1}^{III}\left(0\right).$$

Applying the Formulas (19)–(20) and (22)–(23) and taking into account potential energy in Formula (17), the following displacement equations were obtained:

(26) $$y_{i}^{IV}\left(x_{i}\right)+k_{i}^{2}y_{i}^{II}\left(x_{i}\right)=0,\;\;\;i=1\ldots n.$$

The general solution of Equation (26) is given by the formula

(27) $$y_{i}\left(x_{i}\right)=D_{i1}\sin\left(k_{i}x_{i}\right)+D_{i2}\cos\left(k_{i}x_{i}\right)+D_{i3}x_{i}+D_{i4},$$

where *D_ik_* are integration constants (*κ* = 1, …, 4).

Based on the solutions (27) and the appropriate boundary conditions (19)–(25), a system of homogeneous formulas was determined, which in matrix form can be written as:

(28) $$K_{s}\Lambda =0,$$

where $\Lambda ={\left[C_{11}C_{12}C_{13}C_{14}\ldots C_{n1}C_{n2}C_{n3}C_{n4}\right]}^{T},$$K_{s}$ denotes a square matrix of degree equal to four times the number n of column segments under consideration.

Thus, the following is obtained:

(29) $$K_{s}=\left[\left.\frac{\begin{array}{ccc} M_{0} & 0 & 0\\ H_{1}^{1} & H_{2}^{0} & 0\\ 0 & H_{2}^{1} & H_{3}^{0} \end{array}}{\begin{array}{ccc} 0 & \;0\; & 0\\ 0 & \;0\; & 0\\ 0 & 0 & 0 \end{array}}\right|\frac{\begin{array}{ccc} 0 & 0 & 0\\ 0 & 0 & 0\\ 0 & 0 & 0 \end{array}}{\begin{array}{ccc} H_{n-1}^{1} & H_{n-1}^{0} & 0\\ 0 & H_{n-1}^{1} & H_{n}^{0}\\ 0 & 0 & M_{n} \end{array}}\right],\;\;\;M_{0}=\left[\begin{array}{l} \begin{array}{cccc} 0 & 1 & 0 & 1 \end{array}\\ \begin{array}{cccc} k_{1} & 0 & 1 & 0 \end{array} \end{array}\right],$$

and

(30) $$\begin{array}{l} H_{\zeta +1}^{0}=\left[\begin{array}{cccc} 0 & -1 & 0 & -1\\ -k_{\zeta +1} & 0 & -1 & 0\\ 0 & \chi_{\zeta +1}k_{\zeta +1}^{2} & 0 & 0\\ \chi_{\zeta +1}k_{\zeta +1}^{3} & 0 & 0 & 0 \end{array}\right],\;\;\;H_{\zeta }^{l}=\left[\begin{array}{cccc} \sin(k_{\zeta }l) & \cos(k_{\zeta }l) & l & 1\\ k_{\zeta }\cos(k_{\zeta }l) & -k_{\zeta }\sin(k_{\zeta }l) & 1 & 0\\ -k_{\zeta }^{2}\sin(k_{\zeta }l) & -k_{\zeta }^{2}\cos(k_{\zeta }l) & 0 & 0\\ -k_{\zeta }^{3}\cos(k_{\zeta }l) & k_{\zeta }^{3}\sin(k_{\zeta }l) & 0 & 0 \end{array}\right],\\ \;\;\;\;\;\;\;\;\;\;\;\;\;\;\;\;\;\;\;\;\;\;\;\;\;\;\;\;\;\;\;\;\;\;\;\;\;\;\;\;\;M_{n}=\left[\begin{array}{cccc} m_{11} & m_{12} & m_{12} & m_{14}\\ m_{21} & m_{22} & m_{23} & m_{24} \end{array}\right]. \end{array}$$

The matrix coefficient **M***_n_* is given by the following:

(31) $$\begin{array}{l} m_{11}=-{k_{n}}^{3}\cos\left(k_{n}l\right)+\frac{{k_{n}}^{2}\sin\left(k_{n}l\right)}{R_{1}},\;m_{12}={k_{n}}^{3}\sin\left(k_{n}l\right)+\frac{{k_{n}}^{2}cos\left(k_{n}l\right)}{R_{1}},\;\\ m_{13}=0,\;m_{14}=0,\;m_{21}=\sin\left(k_{n}l\right)-R_{1}k_{n}\cos\left(k_{n}l\right),\;\\ m_{22}=cos\left(k_{n}l\right)+R_{1}k_{n}\sin\left(k_{n}l\right),\;m_{23}=l-R_{1},\;m_{24}=1. \end{array}$$

The value of critical load may be calculated from the transcendental equation as follows:

(32) $$det\;K_{S}=0.$$

## 5. Result of the Free Vibration Issue Considerations

The free vibration frequency ω variability, in a relation to the external load *P* of the systems, was obtained using the solution of the boundary problem with the vibration method (kinetic criterion of loss of stability). By separating the variables with respect to *t* and *x_i_* (see the Formula (18)), the equations of motion (13) may be written in the following form:

(33) $$y_{i}^{IV}\left(x_{i}\right)+k_{i}^{2}\;y_{i}^{II}\left(x_{i}\right)-\Omega_{i}^{2}y_{i}\left(x_{i}\right)=0,\;\;\;\;\;\;\;\;i=1\ldots n.$$

The solutions for Equation (33) are

(34) $$y_{i}\left(x_{i}\right)=C_{i1}\cosh\left(\alpha_{i}x_{i}\right)+C_{i2}\cos\left(\beta_{i}x_{i}\right)+C_{i3}\sinh\left(\alpha_{i}x_{i}\right)+C_{i4}\sin\left(\beta_{i}x_{i}\right),$$

where *Cκ_i_* are integration constants (κ = 1, …, 4) and

(35a) $$\alpha_{i}^{2}=\sqrt{-0.5k_{i}^{2}+\sqrt{0.25k_{i}^{4}+\Omega_{i}^{2}}},$$

(35b) $$\beta_{i}^{2}=\sqrt{0.5k_{i}^{2}+\sqrt{0.25k_{i}^{4}+\Omega_{i}^{2}}},$$

(35c) $$\Omega_{i}^{2}=\frac{\left(\rho A_{i}\right)\omega^{2}}{\left(EJ_{i}\right)},$$

(35d) $$k_{i}=\sqrt{\frac{P}{\left(EJ_{i}\right)}}.$$

After applying (34) to the relevant geometric and continuity boundary conditions, a system of homogenous equations was obtained.

The transcendental equation for the neutral vibration frequency ω of the considered systems can be obtained by equating the determinant of the coefficient matrix **K_D_** to zero, that is

(36) $$K_{D}\;col\left\{G_{11}\;G_{21}\;G_{31}\;G_{41}\;\ldots \;G_{1n}\;G_{2n}\;G_{3n}\;G_{4n}\right\}=0.$$

## 6. Simulated Annealing Method

The simulated annealing approach involves the use of a heuristic algorithm belonging to the class of nondeterministic algorithms. The operation of the algorithm requires the specification of the following parameters: the initial result representation, the generator of random result changes (neighborhood function), the annealing schedule, and the evaluation function (cost). The parameter Θ called “temperature” is an additional parameter that is not associated with the variables being optimized. It affects the performance of the algorithm by changing the probability of transition from one point in the search space to another, and is chosen depending on the given optimization problem.

**Figure 2:**
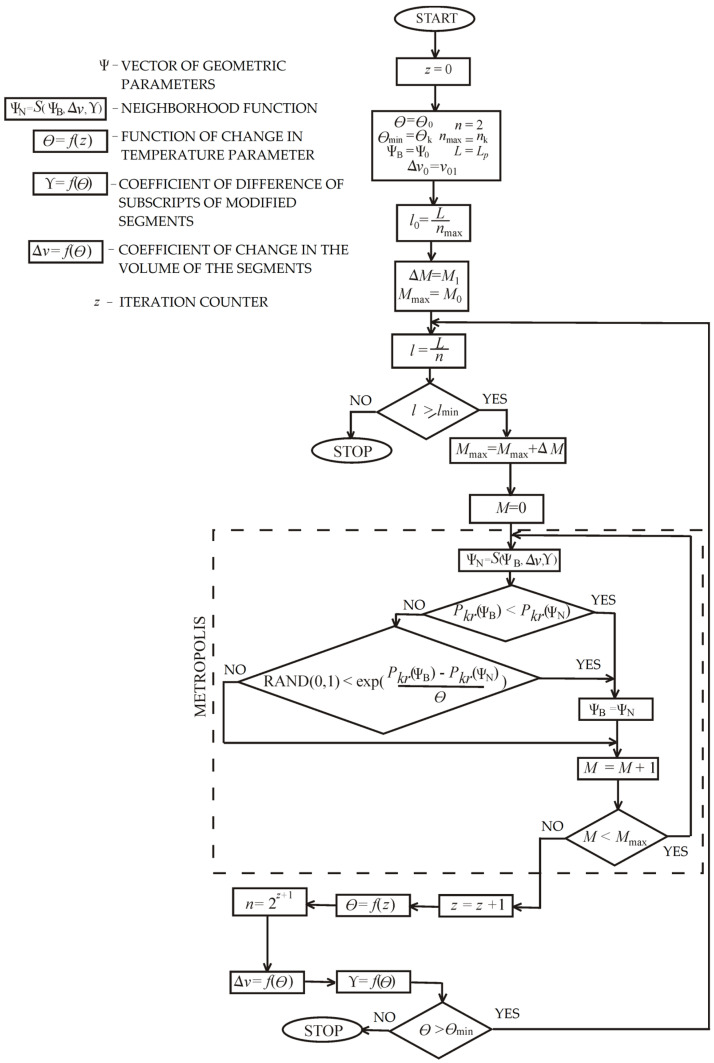
A scheme of the modified simulation annealing algorithm.

The flowchart of the adapted method of simulated annealing is presented in Figure 2. The new result Ψ_N_, obtained by changing the current solution Ψ_B_, is acceptable if the value of the result evaluation function increases. Moreover, acceptance occurs with some probability equal to

(37) $$p=\exp\left(-\frac{\Delta f}{\Theta }\right),$$

where Δ*f* is the change between the values of the evaluation function (Equation (36)) and

(38) $$\Psi =\left[\begin{array}{ccccccc} d_{1} & d_{2} & \ldots & d_{n-2} & d_{n-1} & d_{n} & \frac{L}{n} \end{array}\right].$$

**Figure 3:**
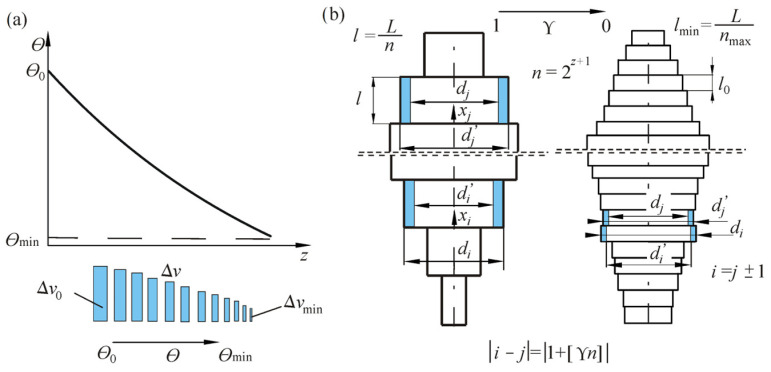
Modifications of the simulated annealing algorithm: (**a**) the “temperature” parameter and (**b**) the neighborhood function.

The Metropolis mode is the core of the algorithm. In this procedure, a series of iterations given by the *M* coefficient were performed with the same “temperature” Θ. Then, the “temperature” was lowered in accordance with the “annealing schedule” which results from the formula Θ = *f*(*z*) (comp. Figure 3a). The amount of iterations *M*_max_ in the next run of the Metropolis mode had increased by a specific value Δ*M*.

In the proposed approach of simulated annealing, an authorial alteration of the function *S*(Ψ_B_) was assumed. In the case under consideration, the operation of the neighborhood function is strongly dependent on the “temperature” parameter (Figure 3).

The proposed approach based on selecting new dimensions relies on “transferring” a specific part of the volume, given by the Δ*v* factor, from a randomly chosen section (described by subscript *i*) to a different section of the rod (described by subscript *j*), also selected randomly (Figure 3b). It is achieved by replacing the dimensions of the chosen sections (*d_i_*, *d_j_*) with the new values of diameters (*d_i_’*, *d_j_’*). The fixed column volume criterion is met thanks to the unchanged lengths of the segments. New values of the diameters of egments are described by the formulas

(39) $$d_{i}^{\prime }=\sqrt{\left(1-\Delta v\right)d_{i}},d_{j}^{\prime }=\sqrt{d_{j}+\Delta vd_{i}},$$

where *i*, *j* ∈ (0…*n*) are the subscripts of the parts of the rod under alterations, and Δ*v* ∈ (0…1) is the coefficient of change in the volume.

The selection of the segment at the initial stage of optimal shaping is completely random. A starting value of Δ*v*_0_ was adopted at the initial stage of the computation, taking into consideration the coefficient of volume change Δ*v*. These values decreased with decreasing parameter, until the minimum Δ*v*_min_ (determined at the initial stage) was met at Θ_min_ (Figure 3).

The parameters Δ*v*, Y, *Θ*, and *M* were changed after each execution of the Metropolis mode. The neighborhood function is given as follows:

**Ψ**_N_ = *S*(**Ψ**_B_, Δ*v*, Y). (40)

The change in the amount of parts *n* and the value of the mentioned parameters are additional implemented modifications of the algorithm of simulated annealing:

(41) $$n=2^{z+1}.$$

Each column segment is bisected into two sections of equal length. The number of parts *n* changes in a geometric progression with a ratio of two to the value *n*_max_ (the maximum amount of parts). The bisection was performed after each stepped loop of Metropolis procedure, as long as the particular segment length *l* was not less than the assumed minimum:

(42) $$l_{\min}=\frac{L}{n_{\max}}.$$

The maximum value of the number of segments was adopted in preliminary studies, taking into account the accuracy of the obtained results and the calculation time. The values of the critical force with *n* = 128 were very close to the system, with twice as many segments (difference in values by a maximum of 0.11%), while the calculation time was much shorter. The numerical values of the algorithm hyperparameters are presented in Table 1.

Thanks to implementation of the described modifications of the algorithm, a significant acceleration in optimal shaping was achieved. After all calculations were performed, due to the small value of the length of the segment, a smooth shape for the system was observed.

## 7. Numerical Computation and Experimental Research

In this section, the numerical computation results, in accordance with the adopted energetic and vibrations methods, and the experimental results, are presented. In the selected case of a Tomski’s load, with selected values of the geometrical parameters of the loading and receiving head radiuses *R*_1o_*, appropriate results from theoretical and numerical studies are shown.

The dependency between the critical load of the systems under optimization and their shape were determined based on the energy method, adopting the condition of the constant volume of the column. The variability of free vibrations as a relation to the external loading and the vibration mode were determined by finding the solution to the boundary problem using the kinetic criterion of stability loss. Experimental studies were carried out within the range of free vibration frequencies.

In the considered case of external load, numerical computation was performed to obtain the maximum of the critical force *P_kr_* of the systems, taking into account the transcendental equation for the critical value (see Formula (32)). It was assumed that the numerical calculations carried out with the division of the rod into *n* = 128 parts are sufficiently accurate. In order to validate the obtained results, the values of the buckling load of the system O(*R*_1o_*j) and the parameters of the loading system were referred to the constant length of the column *L* and the bending stiffness (*EJ*)*_p_* of the relative system P(*R*_1o_*j).

**Figure 4:**
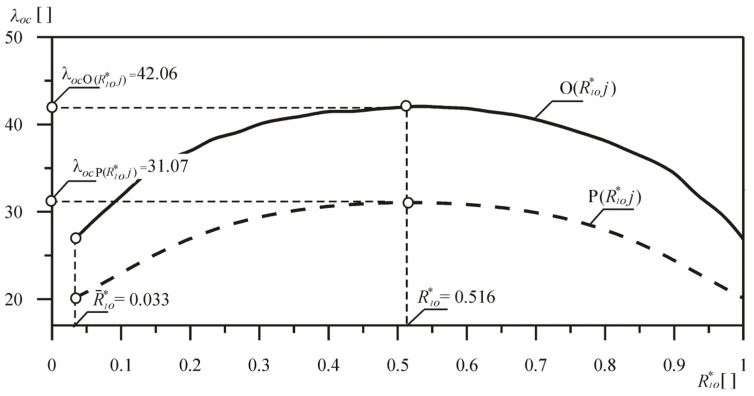
Change in the critical load parameter λ*_oc_* in relation to the value of the parameter *R*_1o_*—columns O(*R*_1o_*j) and P(*R*_1o_*j).

The range of changes in the buckling load parameter of the considered rod, in relation to the changes in the parameters of the loading and receiving head radius, is presented in Figure 4. The continuous lines refer to the results of numerical calculations of the systems with the optimized shape O(*R*_1o_*j), while the dashed lines refer to the corresponding comparative columns P(R_o_*j) with constant bending stiffness.

In the entire range of the loading head dimensions under consideration, for each of the curves, the buckling load coefficient λ*_oc_* maximum was determined. For relative systems, the maximum was achieved with the parameter *R*_1o_*= 0.5. The calculations took into account the range of the load head radius *R*_1_ values $R_{1o}^{*}\in \langle 0,\;1\rangle$. If *R*_1o_* = 0, we obtain the Euler’s load.

The percentage increase in the value of the buckling load δ*_o_* of the rod under optimal shaping is given by the following formula:

(43) $$\delta_{o}=\frac{\lambda_{ocO(R_{1o}^{*}j)}-\lambda_{ocP(R_{1o}^{*}j)}}{\lambda_{ocP(R_{1o}^{*}j)}}\cdot 100\%.$$

**Figure 5:**
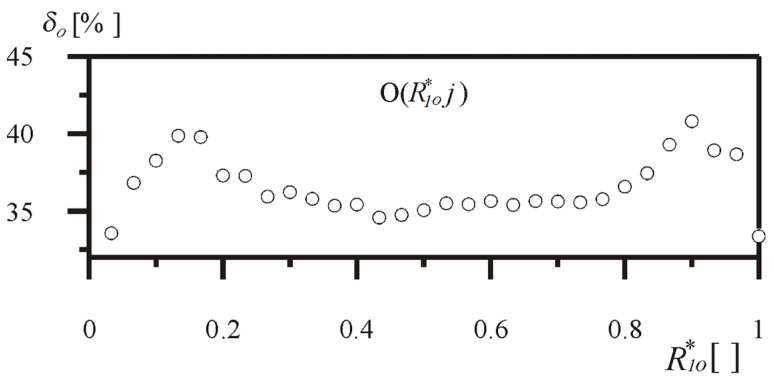
The percentage increase in the value of buckling load δ*_o_* as a function of parameter *R*_1o_^*^—column O(*R*_1o_^*^j).

For each value of the dimensionless parameter of the head radius implementing a load from the tested range, the percentage increase in the critical load was determined in comparison to the prismatic reference system. As shown in Figure 5, taking into consideration division into *n* = 128 segments, the increase in the value of buckling load was achieved by 40.64% at most. 

**Figure 6:**
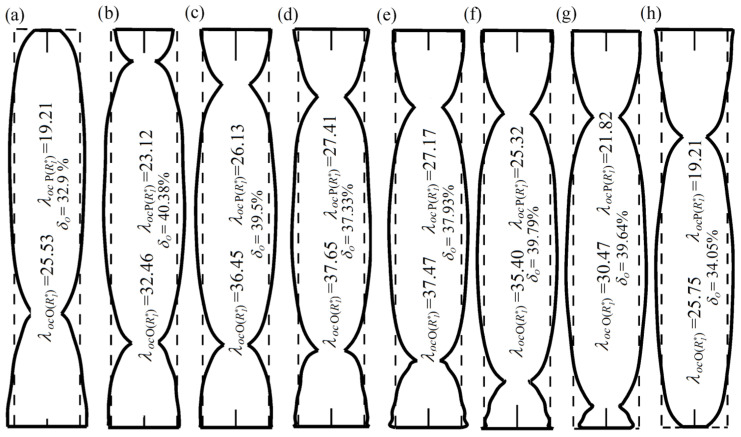
The outlines of the column after optimal shaping O(*R*_1o_^*^j) for different values of the loading and receiving head radiuses *R*_1o_^*^j: (**a**) *R*_1o_^*^ = 0.33, (**b**) *R*_1o_^*^ = 0.433, (**c**) *R*_1o_^*^ = 0.53, (**d**) *R*_1o_^*^ = 0.63, (**e**) *R*_1o_^*^ = 0.73, (**f**) *R*_1o_^*^ = 0.83, (**g**) *R*_1o_^*^ = 0.93, and (**h**) *R*_1o_^*^ = 1.0.

The outlines of the optimized models of rods (optimum material distribution), for chosen values of geometrical dimensions of loading structure, are presented in Figure 6. The columns were built from parts of a constant dimension *d_i_* and a constant length *l* (see Figure 1). The shapes obtained as a result of numerical calculations are “stepped”, but they were approximated by polynomials of a relevant degree and were drawn as if smooth thanks to the significant number of parts. The dashed lines in the picture refer to the outline of the prismatic (comparative) rods. The critical force values of both optimized and comparative columns were both given with the percentage increase in the buckling load for every chosen value of the parameter *R*_1o_^*^. The presence of narrowness in the outlines is a characteristic feature for all presented shapes and its locations depend on the geometric parameters of loading structure that are involved in the discussed external force.

Numerical calculations were performed with reference to the natural frequency ω of the O(*R*_1o_^*^j) column, taking into account the solution of the boundary value problem presented in Section 5 of this paper. Taking into account the variable material distribution, the studies were limited to determining the variability of the first two natural frequencies in dimensionless form (*Ω_o_*_1_, *Ω_o_*_2_), in relation to the dimensionless load parameter λ*_o_*, for selected values of R_o_^*^, Δ*r* of the heads realizing the specific load. The following is assumed,

(44) $$\;\;\lambda_{o}=\frac{PL^{2}}{{\left(EJ\right)}_{p}},$$

(45) $$\;\Omega_{ο}=\frac{{\left(\rho A\right)}_{p}\omega^{2}L^{4}}{{\left(EJ\right)}_{p}},$$

where

(ρ*A*)*_p_*—compressive stiffness of the comparative column.

**Figure 7:**
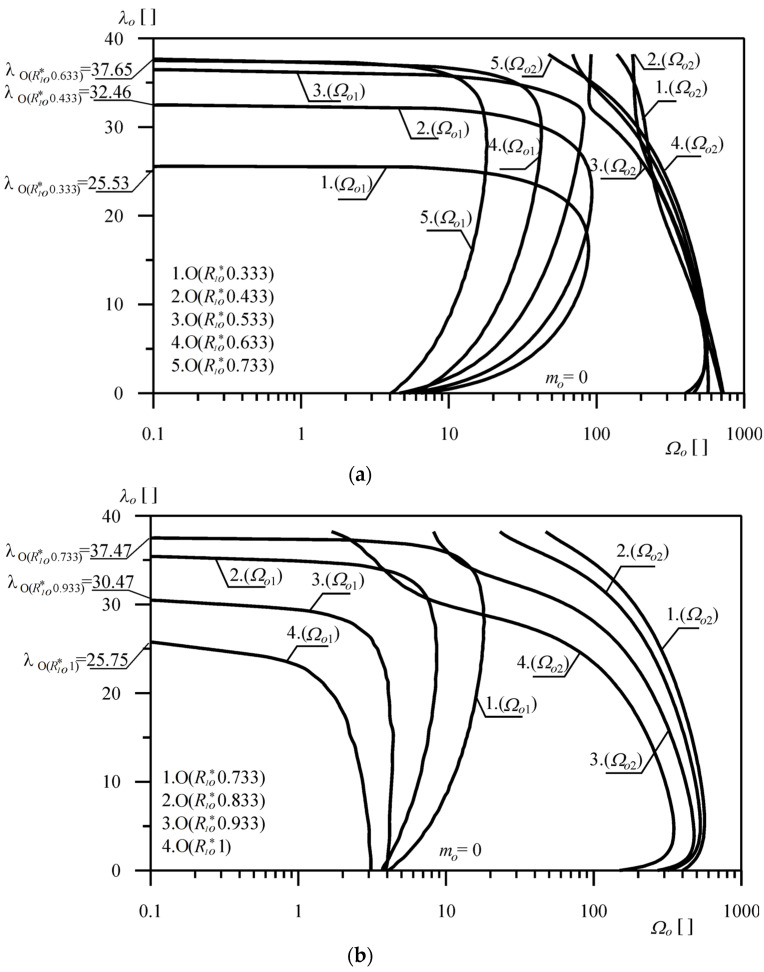
(**a**) Change in the natural frequency parameter Ω*_o_* as a function of the external load parameter λ*_o_* (O(R_o_^*^j) system). (**b**) Change in the natural frequency parameter Ω*_o_* as a function of the external load parameter λ*_o_* (O(R_o_^*^j) system).

Figure 7a,b show the results of the numerical computation of the variability of the natural frequencies of the O(*R*_1o_^*^j) column. In the case of *R*_1o_^*^ = 0 (curves 1—Figure 7a), the column corresponds to the Euler load case. Studies were carried out for selected values of the dimensionless parameter of the loading head radius.

**Figure 8:**
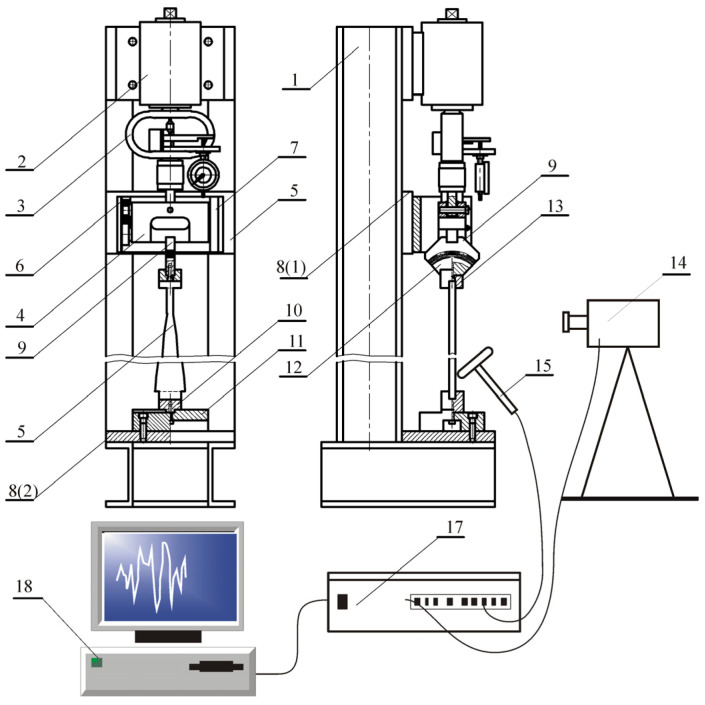
Stand for testing free vibrations of vertically positioned columns.

Figure 8 shows the change in the first frequency of the O(*R*_1o_^*^j) and P(*R*_1o_^*^j) columns, with the given head parameter *R*_1o_^*^. In the numerical computation, the mass *m* was assumed to be zero, where

(46) $$m_{o}=\frac{m}{{\left(\rho A\right)}_{p}L}.$$

The value of the external load at which the parameter Ω*_o_*_1_ = 0 corresponds to the critical load of the discussed columns. The correctness of the adopted models and assumptions is proven here by the agreement of the critical load obtained on the basis of the kinetic and static stability loss criterion.

The presented numerical studies were verified by experimental studies. The dimensions and physical properties of the analyzed column and the load-receiving head are given in Table 2.

For the calculations and experimental tests, it was assumed that the cross-section of the analyzed rod is a rectangle of dimensions *a* and *b*. Taking into account the assumed constant volume of the column, the width *a* was determined with its fixed thickness *b*. In the proposed optimization approach, an extra condition was taken into account:

(47) $$a_{i}\geq b+0.001\;\;\;\;[m]\;\;\;\;\;i=1,\ldots ,n$$

The limitation of the geometric inequality (47) of the rod under optimal shaping is excused because of the plane of buckling for the column, given by the minimum moment of inertia about the neutral axis in the bending plane.

The experimental tests were performed on a stand constructed and built at the Faculty of Mechanics and Machine Design Fundamentals of the Częstochowa University of Technology. The stand is presented in Figure 8. The element of the head (2) is attached to a support frame (1) and is a screw system where the force loading the column under consideration (5) is generated by the dynamometer (3) and a plate (4). The required boundary conditions of the column are implemented by supports attached to plates 8 (1) and 8 (2). A linear feed in the rails (7) attached to plate 8 (1) is ensured through bearings (6) used at the pivots of the base (4). The boundary conditions at the free end are guaranteed by a loading head (9) attached to the plate (4). The component (1) that is mounted to the base 8 (2) with the housing (11) ensures the condition of rigid bracing of the system. The head receiving the external force (12) is mounted to the rod under consideration with the use of block (13). It is assumed that the parts of the structure receiving external load, (12), (13), and (6), are infinitely rigid, which is dictated by construction considerations. Figure 8 also shows a diagram of the measurement system that consists of a laser vibrometer OMETRON (14) (model VH–1000–D), a modal hammer Brüel&Kjaer (15) (8200 + 2646 type), an analyzer Brüel&Kjaer (17) (3560C type), and a computer (18) with PULSE software.

As part of numerical calculations, the outline of the rod under optimal shaping was also determined. Taking into consideration the parts that are assumed to be infinitely rigid, (12) and (13), of the load-receiving head in the free vibration problem formulation, the boundary conditions Formulas (20) and (21) are modified. In this way, the following dependencies are obtained:

(48) $$\left\{\begin{array}{l} Y_{n}\left(l,t\right)-\left(R_{1}-l_{0}\right){\left.\frac{\partial Y_{n}\left(x_{n},t\right)}{\partial x_{n}}\right|}^{x_{n}=l}=0,\\ {\left.\frac{\partial^{3}Y_{n}\left(x_{n},t\right)}{\partial x_{n}^{3}}\right|}^{x_{n}=l}-\frac{1}{\left(R_{1}-l_{0}\right)}{\left.\frac{\partial^{2}Y_{n}\left(x_{n},t\right)}{\partial x_{n}^{2}}\right|}^{x_{n}=l}-\frac{m}{\left(EJ_{n}\right)}\frac{\partial^{2}Y_{n}\left(x_{n},t\right)}{\partial t^{2}}=0, \end{array}\right.$$

where *l*_0_—total length of rigid elements (12) and (13)—see Figure 8.

**Figure 9:**
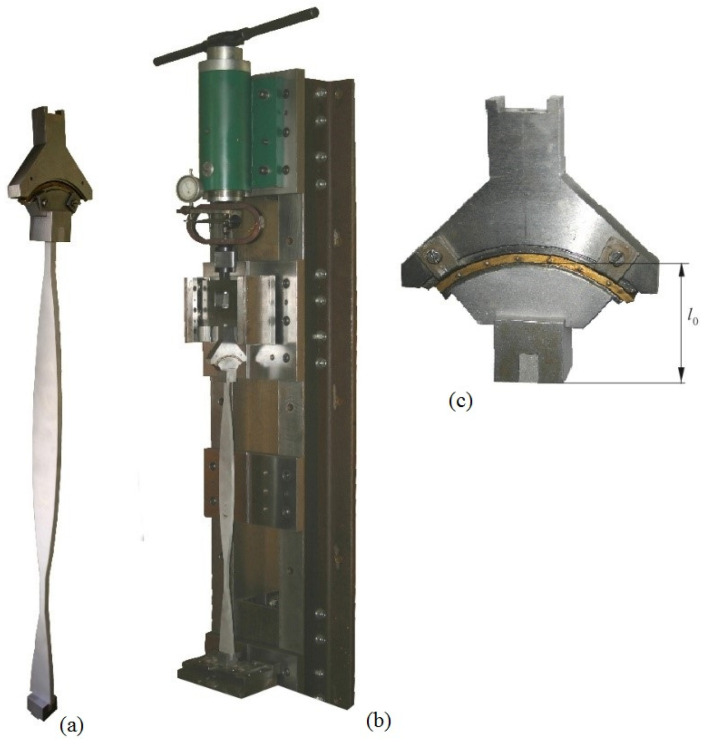
Experimental stand for transverse vibration studies on elastic rods: (**a**) examined structure (**b**) stand with a mounted rod, and (**c**) loading structure.

**Figure 10:**
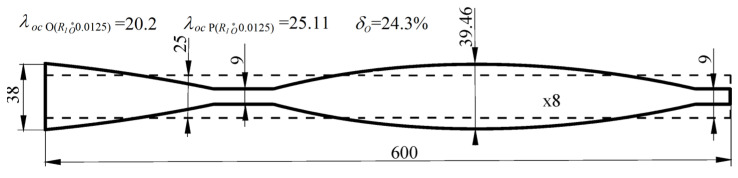
Column outlines: O(*R*_1o_^*^ 0.0125), P(*R*_1o_^*^ 0.0125).

The design solutions of the test stand are shown in Figure 9, where the outline of the systems under optimal shaping and the constructional solution of the loading structure are also shown (Figure 9a,c). The outline of the O(*R*_1o_^*^ 0.0125) column under optimal shaping (continuous lines) is presented in Figure 10. The dashed line shows the shape of the corresponding reference system P(*R*_1o_^*^ 0.0125) column. In the presented case, the received buckling load increase exceeds 24%.

**Figure 11:**
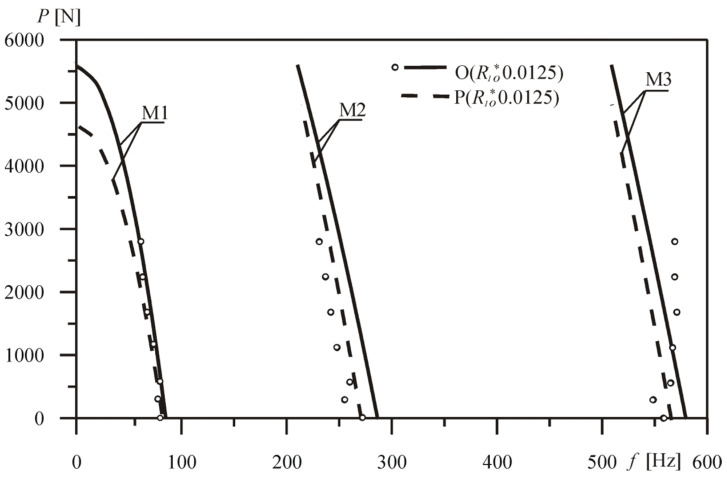
Curves on the external force *P*—free vibration frequency *f* (O(*R*_1o_^*^ 0.0125)) plane.

The comparison of the first three natural frequencies *f* depending on the external force *P*, obtained for the optimized system and the comparative prismatic system, is shown in Figure 11. Changes in the eigenvalues of the comparative rod P(*R*_1o_^*^ 0.0125) are presented by dashed lines. By comparing the numerical and experimental results with the reference system (column O(*R*_1o_^*^ 0.0125)), the authors conclude that there is good consistency between the models mentioned. The maximum value of the relative error recorded for the first frequency between the experimental results and the theoretically obtained values was 7.24%.

The experimental study could only be performed within a certain range of external load, because at values close to the critical load, the system stiffens and the obtained results are inadequate for the boundary conditions. The direction of the external load strictly depends on the geometry of the head that implements the load. These heads are made of rolling bearing segments, which ensure the tracking of the free end of the column and meet the condition of the force directed towards the pole. Verification of numerical studies in the full range of the head radius parameter was therefore difficult.

The sources of the differences in results, apart from measurement inaccuracies, may be simplifications adopted in the mathematical model (the omission of the resistance to movement of the contact between the load-carrying head elements, the assumption of infinite stiffness of the column mounting, and the assumed values of Young’s modulus and the density of the column material), errors in reading from the measuring equipment, or the effects of the test stand.

## 8. Conclusions

The issues of free vibrations and stability in elastic slender columns subjected to the action of a follower force directed towards the positive pole, subjected to theoretical, numerical, and experimental analysis, are presented in this article. The principle of minimum potential energy was used to formulate the problem. Considering the variable material distribution along the column axis (non-prismaticity), a transcendental equation allowing for buckling load calculations was derived, which is the objective function in the optimal shaping problem. A modified simulated annealing algorithm was proposed. The introduction of the proposed modifications to the simulated annealing algorithm significantly accelerates the shape optimization process, especially at the initial stage of calculations when determining the “rough” outline of the column. In the final phase, with a much larger number of segments, the shape change smooths the outline. Assuming a constant volume of the systems, analyzing the variable material distribution, the buckling load increased in the entire tested range of the dimensionless parameter of the head radius, reaching a percentage increase of over 40% compared to the reference systems. It was shown that there is such a geometry of the load-bearing system and the column, for which the maximum buckling load was achieved. As a result of the optimization, it was noted that each of the obtained column shapes is characterized by the occurrence of cross-sectional narrowings, the location of which along the axis depends on the values of the geometrical parameters of the system. Based on the obtained improvement of the strength properties of the column, it was proved that the adopted modified simulated annealing approach is an efficient procedure for optimizing the shape of slender systems, even if the objective function is a function of many variables.

In relation to the vibration method, based on Hamilton’s principle, appropriate equations of motion and boundary conditions were derived. In order to determine the vibration frequency variability of the columns under optimal shaping in relation to the external force, a series of numerical calculations were performed. The results are presented in the plane: load parameter *λ*_o_—natural frequency parameter *Ω*_o_ and natural vibration modes. The results concerning the critical forces obtained from the static and kinetic stability loss criterion are consistent, which proves the correctness of the construction of mathematical models. The correctness of the adopted mathematical model in the range of free vibrations was also verified by experimental studies, analyzing the change in the vibration frequency depending on the external load and vibration modes, with the adopted geometric parameters. The results obtained from numerical calculations and experimental studies are similar, and the maximum relative error of the vibration frequency was about 7%. The authors indicated potential reasons for the differences in the obtained results: measurement inaccuracies, simplifications adopted in the mathematical model, errors in reading from the measuring equipment, or the effects of the test stand.

## Figures and Tables

**Table 1:** The values of some parameters of the optimization algorithm.

Initial Temperature Value	Minimum Temperature Value	Cooling Coefficient	Maximum Number of Segments
50	0.05	0.2	128

**Table 2:** Parameters of the system under optimal shaping O(*R*_1o_^*^j).

*E* [Pa]	*ρ* [kg/m^3^]	*L* [m]	*b* [m]	*R*_1_ [m]	*l_o_* [m]	*m* [kg]
7.5 × 10^10^	2790	0.6	0.008	0.059	0.051	0.39

## Data Availability

The original contributions presented in this study are included in the article and further inquiries can be directed to the corresponding author.

## Nomenclature

*a_i_,b*	dimensions of the *i*-th segment of the optimized column
*A_i_*	cross-sectional area of the *i*-th segment of the optimized column
*d*	diameter of the prismatic comparative column
*E*	Young’s modulus
*J_i_*	moment of inertia of the cross-section of the *i*-th segment of the optimized column
*l*	length of the *i*-th segment of the optimized column
*l*_0_	geometrical parameter of the loading structure
*L*	total length of the column
*m*	concentrated mass
*m*_0_	dimensionless parameter of concentrated mass
*n*	number of segments
*P*	external load
*P_kr_*	critical force
*R*_1_	loading head radius
*R*_10_ *^*^*	loading head radius dimensionless parameter
***t***	time
***T***	kinetic energy
***v_i_***	volume of the *i*-th segment of the optimized column
***V***	total volume of the column
***V*****_1_**, ***V*****_2_**, ***V*****_3_**	potential energy
x	generalized coordinate
(*EJ_i_*)	flexural stiffness of the *i*-th segment of the optimized column
(*EJ*)*_pr_*	flexural stiffness of the prismatic comparative column
Y*_i_*(*x_i_,t*) y*_i_*(*x_i_*)	transverse displacement of the *i*-th segment of the optimized column
(ρ*A_i_*)	mass per unit length of the *i*-th segment of the optimized column
(ρ*A*)*_p_*	mass per unit length of the comparative column
*δ*	symbol of the variation
*δ_o_*	percentage increase in the value of critical load
$\lambda_{oc}$	dimensionless parameter of critical force of the optimized column
*ρ*	density of the column material
ω	natural frequency
Ω*_o_*_i_	dimensionless parameter of the *i*-th natural frequency

## References

[1] Wagg D., Neild S. (2015). Introduction to Nonlinear Vibration and Control. Nonlinear Vibration with Control for Flexible and Adaptive Structures.

[2] Yaghoobi H., Torabi M. (2013). An analytical approach to large amplitude vibration and post-buckling of functionally graded beams rest on non-linear elastic foundation. J. Theor. Appl. Mech..

[3] Elmaguiri M.N., Haterbouch M., Bouayad A., Oussouaddi O. (2015). Geometrically nonlinear free vibration of functionally graded beams. J. Mater. Environ. Sci..

[4] Kumar S., Mitra A., Roy H. (2015). Geometrically nonlinear free vibration analysis of axially functionally graded taper beams. Int. J. Eng. Sci. Technol..

[5] Ruilan T., Xinwei Y., Zhang Q., Xiuying G. (2020). Vibration reduction in beam bridge under moving loads using nonlinear smooth and discontinuous oscillator. Adv. Mech. Eng..

[6] Akour S.N. (2012). Parametric Study of Nonlinear Beam Vibration Resting on Linear Elastic Foundation. J. Mech. Eng. Autom..

[7] Giżejowski M.A., Szczerba J., Gajewski M.D., Stachura Z. (2017). Buckling resistance assessment of steel I-section beam-columns not susceptible to LT-buckling. Arch. Civ. Mech. Eng..

[8] Szmidla J., Jurczyńska A. (2020). Local loss of a rectilinear form of a static equilibrium of geometrically nonlinear system with non-prismatic element under force directed towards the pole. Acta Phys. Pol. A.

[9] Nikolic A., Salinic S. (2017). Buckling analysis of non-prismatic columns: A rigid multibody approach. Eng. Struct..

[10] Serna M.A., Ibanez J.R., Lopez A. (2011). Elastic flexural buckling of non-uniform members: Closed-form expression and equivalent load approach. J. Constr. Steel Res..

[11] Das D., Ayoub A. (2021). Mixed formulation for geometric and material nonlinearity of shear-critical reinforced concrete columns. Eng. Struct..

[12] Adorno R., Palazotto A.N. (2023). A finite element study on the effects of thickness and material nonlinearity on the equilibrium path of axially loaded circular cylindrical shells. Eng. Struct..

[13] Liu T., Sun X., Hu W., Wang L., Zhang S., Bui T.Q. (2024). Nonlinear thermal-mechanical coupled isogeometric analysis for GPLs reinforced functionally graded porous plates. Eng. Struct..

[14] Jiang J., Ke L. (2024). Linear and nonlinear vibrations of nonlinearly elastically constrained functionally graded porous microbeams with rough surface. Eng. Struct..

[15] Eiadtrong S., Nguyes T.N., Wattanasakulpong N. (2024). Nonlinear vibration of sandwich beams made of FGM faces and FGP core under multiple moving loads using a quasi-3D theory. Eng. Struct..

[16] Jurkiewiec B., Darwich H., Grazide C. (2024). Behaviour of GFRP-timber hybrid beams in torsion and bending. Eng. Struct..

[17] Sun H., Chen J. (2024). Vibration reduction of graphene reinforced porous nanocomposite beams under moving loads using a nonlinear energy sink. Eng. Struct..

[18] Zhang Y., Guo X., Wu Y., Zhang Y., Zhang H., Chaofeng L. (2024). Vibration control of membrane structures by piezoelectric actuators considering piezoelectric nonlinearity under strong electric fields. Eng. Struct..

[19] Gong F., Xia Y., Lozano F., Yu B. (2024). Experimental analysis of structural nonlinear damping ratio induced by bolt joint friction. Eng. Struct..

[20] Uzny S., Sokół K. (2015). Critical Load of a Differently Mounted Columns Determinated According to the Bernoulli-Euler and Timoshenko Theories. Mach. Dyn. Res..

[21] Tomski L., Uzny S. (2011). A hydraulic cylinder subjected to Euler’s load in aspect of the stability and free vibrations taking into account discrete elastic elements. Arch. Civ. Mech. Eng..

[22] Przybylski J., Sokół K. (2011). Shape control of an eccentrically loaded column by means of a piezoceramic rod. Thin-Walled Struct..

[23] Szmidla J., Jurczyńska A. (2015). Stability of Geometrically Nonlinear Pre-stressed Column Loaded by a Force Directed Towards the Positive Pole Partially Lying on Winkler Elastic Foundation. AIP Conf. Proc..

[24] Colmenares D., Andersson A., Karoumi R. (2021). Closed-from solution for mode superposition analysis of continuous beams on flexible supports under moving harmonic loads. J. Sound. Vibr..

[25] Apsemetov M., Madanbekov N., Muktarov T. (2021). Determination of the own forms of vibration of the span of beam bridges on elastic supporting parts. E3S Web Conf..

[26] Zhang X., Wang Y., Liu M., Cao Y., Chen S., Cao D. (2024). Nonlinear dynamical modeling and response analysis of complex structures based on assumed mode weightinh. Eng. Struct..

[27] Li Q.S. (2001). Exact solutions for buckling of non-uniform columns under axial concentrated and distributed loading. Eur. J. Mech.-A/Solids.

[28] Li Q.S. (2008). Stability of non-uniform columns under the combined action of concentrated follower forces and variably distributed loads. J. Constr. Steel Res..

[29] Szmidla J., Jurczyńska A. (2015). The Tapered Column Shape Optimization in a Plane Perpendicular to the Buckling Plane Subjected to a Load by the Follower Force Directed to the Positive Pole. Mach. Dyn. Res..

[30] Da Silva L.A.R., Torri A., Beck A.T. (2024). System-reliability-based sizing and shape optimization of trusses considering millions of failure sequences. Struct. Safe.

[31] Wahrhaftig A.d.M., Magalhaes K.M.M., Silva M.A., da Fonseca Brasil R.M.L.R., Banarjee J.R. (2022). Buckling and free vibration analysis of non-prismatic columns using optimized shape functions and Rayleigh method. Eur. J. Mech.-A Solids..

[32] Du X., Li J., Wang W., Zhao G., Liu Y., Zhang P. (2024). Isogeometric Shape Optimization of Reissner-Mindlin Shell with Analytical Sensitivity and Application to Cellular Sandwich Structures. Comp.-Aid. Design.

[33] Zhou G., Qian C., Chen C. (2022). Research on the Approximate Calculation Method of the Fundamental Frequency and Its Characteristics and a Tensioned String Bridge. Processes.

[34] Garinei A. (2006). Vibrations of simple beam-like modelled bridge under harmonic moving loads. Int. J. Eng. Sci..

[35] Raspopov A., Artyomov V., Rusu S. (2010). The Simulation of Vibrations of Railway Beam Bridges in the Object-oriented Environment Delphi. Arch. Transp..

[36] Zangeneh A., Museros P., Pacoste C., Karoumi R. (2021). Free vibration of viscoelastically supported beam bridges under moving loads: Closed-form formula for maximum resonant response. Eng. Struct..

[37] Doungporn W., Nathnarong K., Yong H.W. (2019). Effect of beam joinery on bridge structural stability. Adv. Differ. Equ..

[38] Delahaye D., Chaimatanan S., Mongeau M., Gendreau M., Potvin J.-Y. (2019). Simulated annealing: From basics to applications. Handbook of Metaheuristics.

[39] Guilmeau T., Chouzenoux E., Elvira V. Simulated annealing: A review and a new scheme. Proceedings of the SSP 2021—IEEE Statistical Signal Processing Workshop.

[40] Soonjun B., Krityakierne T. (2024). GDESA: Gradient Differential Evolution-Simulated Annealing Hybrid. IEEE Access.

[41] Mateev V., Marinova I. Optimization of Heat Sink Design by Simulated Annealing Method. Proceedings of the XXXII International Scientific Conference Electronics (ET 2023).

[42] Holzschuh B., Lähner Z., Cremers D. Simulated Annealing for 3D Shape Correspondence. Proceedings of the International Conference on 3D Vision (3DV).

[43] Bulut V. (2024). Design of optimal quasi-developable surface via simulated annealing based shape-parameter-search algorithm. Ann. Math. Artif. Intell..

[44] Brown A., Droge G. (2024). A Simulated Annealing Approach to the Scheduling of Battery-Electric Bus Charging. Future Transp..

